# Comprehensive characterization and translational implications of the *Galns*^R384C^ mouse model of Mucopolysaccharidosis IVA

**DOI:** 10.1038/s10038-026-01501-x

**Published:** 2026-08-13

**Authors:** Dione A. Holder, Betul Celik, Sampurna Saikia, Shaukat A. Khan, Yasuhiko Ago, Amali Karunathilaka, Shunji Tomatsu

**Affiliations:** 1 Department of Biological Sciences, University of Delaware, Newark, DE USA; 2 Department of Biomedical Research, Nemours Children’s Health, Wilmington, DE USA

**Keywords:** Molecular biology, Metabolic disorders, Experimental models of disease

## Abstract

Mucopolysaccharidosis IVA (MPS IVA) is a lysosomal storage disorder caused by a deficiency of N-acetylgalactosamine-6-sulfate sulfatase (GALNS), leading to progressive accumulation of keratan sulfate (KS) and chondroitin-6-sulfate (C6S) and resulting in systemic skeletal dysplasia. Severe, early-onset disease is frequently associated with destabilizing structural missense variants, including p.R386C. To model a loss-of-function missense variant associated with severe MPS IVA, we generated a *Galns*^*R384C*^ knock-in mouse, the murine ortholog of the most common human variant, p.R386C. Biochemical, histological, and skeletal phenotypes were evaluated across multiple tissues, and bone microarchitecture was assessed using microcomputed tomography (micro-CT). Genomic and biochemical assays were performed to assess allelic integrity, and principal component analysis (PCA) was used to integrate biochemical and structural parameters. *Galns*^*R384C*^ mice exhibited significantly reduced GALNS activity and elevated KS levels across various tissues. Histological examination revealed considerable vacuolization in cartilage and cardiac valves, while micro-CT illustrated altered bone microarchitecture consistent with disrupted endochondral ossification. During allele validation, a secondary missense variant (p.R384Y) was identified and characterized as a comparative model that led to defective GALNS activity, substrate accumulation, and analogous skeletal and cardiovascular pathology. PCA demonstrated clear differentiation between WT and mutant groups, with considerable multivariate overlap observed between *Galns*^*R384C*^ and *Galns*^*R384Y*^ mice. In conclusion, *Galns*^*R384C*^ and *Galns*^*R384Y*^ mice recapitulate key biochemical, skeletal, and histopathological features of MPS IVA and provide well-characterized murine models of severe GALNS loss-of-function resulting from clinically relevant missense variants. Rigorous genomic validation underscores the importance of careful allele-level characterization during genome-editing-based model generation.

## Introduction

Mucopolysaccharidosis IVA (MPS IVA, MIM#253000), also known as Morquio A syndrome, is a rare, autosomal-recessive genetic disorder caused by a dysfunctional N-acetylgalactosamine-6-sulfate sulfatase (*GALNS*) gene. In normal physiology, GALNS functions intracellularly to remove sulfate groups from glycosaminoglycans (GAGs) during their catabolism. Pathogenic variants in *GALNS* result in deficient enzyme activity, leading to lysosomal accumulation of undegraded keratan sulfate (KS) and chondroitin-6-sulfate (C6S), particularly in chondrocytes and other connective tissue cells, causing systemic progressive skeletal dysplasia and multi-organ manifestations [1]. Unlike other MPS disorders, MPS IVA is primarily characterized by skeletal dysplasia, including short stature, joint abnormalities, and spinal instability, with little to no neurological involvement [2, 3]. Due to these skeletal manifestations, MPS IVA patients suffer from severe mobility impairments, respiratory complications, and reduced quality of life [4, 5].

Currently, enzyme replacement therapy and hematopoietic stem cell transplantation are clinically available interventions for MPS IVA [6–9], although neither approach fully corrects skeletal pathology. Preclinical studies using viral vectors, stem cell–based approaches, and genome editing have provided important biological insights, but definitive conclusions regarding skeletal rescue remain limited [9–15].

The pathogenesis of MPS IVA is multifactorial, involving complex interactions between genetic predisposition and metabolic regulation. While some rare cases are caused by nonsense or frameshift mutations that produce truncated, nonfunctional proteins, the vast majority of patients harbor missense variants that alter a single amino-acid residue within the GALNS enzyme, frequently perturbing enzyme stability or catalytic function, ultimately resulting in reduced GALNS activity [16, 17].

Animal models, particularly mice, have been instrumental in unraveling the molecular and physiological underpinnings of MPS IVA [18–20]. These models offer controlled environments for understanding disease progression, support mechanistic studies, and provide a foundation for future therapeutic investigations. However, the existing missense-mutant mouse model for MPS IVA focuses on the active-site missense mutation (C76S, the murine ortholog of human C79S), which impairs catalytic function and consistently recapitulates the severe bone pathology phenotype [21]. Although valuable for understanding underlying mechanisms, this model may obscure the more nuanced biological impacts of specific variants observed in patients. To address this issue, the current study concentrates on a singular, clinically representative missense mutation, R386C, which is among the most prevalent and severe human missense mutations associated with the classic, early-onset phenotype observed in numerous countries, including the United States [22–24]. R386C is predicted to disrupt local charge interactions and compromise the stability of the GALNS protein structure [22, 25, 26].

Currently, contemporary murine models frequently utilize CRISPR/Cas9 technology [27]. While this method is highly effective, it presents certain quality-control challenges, including founder mosaicism [28, 29], allele dropout caused by primer-site mismatch [30] and large on-target rearrangements [29] that may escape detection by standard PCR techniques. These limitations can complicate the interpretation of genotype–phenotype correlations if not carefully managed, thereby highlighting the importance of comprehensive validation procedures when interpreting genotype–phenotype relationships [28].

By integrating the utility of murine models with the specificity of a clinically relevant mutation and incorporating a CRISPR-aware validation pipeline, this research is positioned to produce both allele-specific mechanistic insights and applicable findings for the development and interpretation of preclinical models in MPS IVA. Accordingly, this study emphasizes post-establishment allele validation rather than founder-stage phenomena.

## Materials and methods

### Generation of *Galns*^*R384C*^ mouse line

The *Galns*^*R384C*^ mice were generated with a CRISPR/Cas9-induced mutation in exon 11 of the *Galns* gene. Schematic representation of the mouse *Galns* model used in this study is shown in Supplementary Fig. S1. This custom knock-in mouse line was generated by Inotiv (Gaithersburg, MD). The homologous *Mus musculus* line to that of humans was determined (i.e., 384 to 386 humans). A single RNA guide was designed to target exon 11 and drive the Cas9 double-strand break close to the 1152C genomic position. The full target site was 5′-aggcccatattttattaccgTGG-3′ (PAM in uppercase), and the predicted cleavage site for CRISPR-Cas9 is located 3 bp upstream of the PAM. A single-strand donor DNA (ssODN) sequence was designed to introduce both the 1152C>T missense mutation and a new TauI restriction site, which is required for genotyping of the offspring. The ssODN contained homology arms flanking the predicted cleavage site to facilitate repair via homology-directed repair. The donor sequence (5′ → 3′) was:

[AACACACGGACTTGGTCTTCACTCTTTCTCTCCTCAACACAACAGGCCCATATTTTATTAC**TGC**GGCAACACTCTGATGGCTGTCACTCTTGGCCAGTACAAAGCACACCTCTGGACTTGGACC]. Introduced nucleotide substitutions are indicated in bold. The genome editing was performed in zygotes to generate founder mice. A total of 68 mice have been screened with *Galns*Cel1F and *Galns*Cel1R primers, and 6 of them (2 females and 4 males) were identified to be positive for the point mutation R384C. The two founders were backcrossed to wild type (WT) to generate heterozygous (HET) progeny. Additionally, founder mice underwent genomic sequencing, and their R384C mutation was confirmed.

Upon receipt, mice were bred in-house. All mice included in this study were derived from this established line maintained over multiple generations (for ≥3 generations) before phenotypic characterization.

### Mouse colony maintenance

All animal experiments were approved by the Nemours Children’s Health Institutional Animal Care and Use Committee (IACUC: protocol #RSP20-12482-003) and conducted in accordance with all relevant ethical regulations governing animal testing and research. Mice were housed under standard conditions with ad libitum access to food and water and a 12-h light/dark cycle. The colony was maintained through routine breeding strategies over multiple generations before inclusion in this study.

### Genotyping and cohort assignment

All experiments were performed using age-matched homozygous MPS IVA and WT male mice (*n* > 4 per group). All analyses were performed using age-matched male mice to reduce biological variability during initial phenotypic characterization. Mice were euthanized at 16 weeks of age, and tissues were collected for all subsequent analyses (Supplementary Fig. S1B). For genotyping of the knock-in mice, genomic DNA was extracted from tail biopsies/ear clips using standard protocols [12, 31], and PCR was performed to distinguish each genotype. The PCR reaction generated a 439 bp amplicon, a sequence encompassing the point mutation, which was further digested with the TauI restriction enzyme. PCR primer pairs and fragment base pairs corresponding to the genotypes are listed in Supplementary Table S1.

*Galns* locus verification via Sanger sequencing was performed across all 14 exons using primarily intron-flanking primers, with long-range polymerase used for most amplicons. Cohort genotypes were confirmed using the original PCR-RFLP assay, a WT-sequence/site PCR-RFLP confirmation assay, and intronic amplification spanning exon 11 (Intron10F/Intron11R) (Supplementary Figure S2).

### Tissue collection

Blood samples were collected biweekly in EDTA-containing tubes (BD Microtainer^®^; BD, Franklin Lakes, NJ) by venous puncture on the superficial temporal vein with a 22 G needle (BD Eclipse™ Vacutainer^®^; BD), starting at 4 weeks until 16 weeks. Plasma samples were stored at −80 °C until their processing. Additionally, at autopsy, transcardiac perfusion with NaCl 0.9% w/v was performed with a Masterflex L/S Easy-Load II system (Avantor, Radnor, PA) using a 27 G × 3/4 scalp vein (butterfly) needle into the left ventricle. Then, the arms, brain, eyes, gonads, heart, kidneys, legs, liver, lungs, muscle, spine, spleen, and trachea were collected and preserved at -80 °C for biochemical assays and in 4% paraformaldehyde (PFA) for histopathological analysis.

### GALNS enzyme assay

GALNS enzyme activity was measured in plasma and tissues using a 4-methylumbelliferone (4-MU) fluorogenic substrate (Melford Laboratories Ltd, Suffolk, UK) [10, 32]. For assay preparation, plasma was thawed on ice. Tissues (10–50 mg) were homogenized in homogenization buffer (25 mM Tris–HCl, pH 7.2, 1 mM PMSF) using a Bead Ruptor homogenizer (OMNI International, Kennesaw, GA). Homogenates were clarified at 12,000 × *g* for 30 min at 4 °C, and the supernatants were used for enzyme assays. Plasma or tissue lysates were incubated with 4-μM β-galactopyranoside-6-sulfate (Carbosynth, San Diego, CA) in 0.1 M NaCl/0.1 M sodium acetate (pH 4.3) at 37 °C for 16–17 h, followed by a 1-h incubation with β-galactosidase from *Aspergillus oryzae* (Sigma-Aldrich, St. Louis, MO). Reactions were performed in duplicate. Enzymatic activity was terminated with 1 M glycine NaOH buffer (pH 10.5), and fluorescence was measured at Ex 360 nm/Em 460 nm via the FLUOstar Omega plate reader (BMG LABTECH Inc, Cary, NC). The activity was expressed as nanomoles of 4-methylumbelliferone released per hr per milligram of protein (nmol/h/mg). Protein concentrations were determined using a Pierce^TM^ BCA protein assay kit (Thermo Fisher Scientific; Waltham, MA). All fluorescent procedures were performed under low-light conditions to prevent photodegradation.

### GAG quantification

GAGs were extracted from tissues as previously described [10, 32, 33] with minor modifications. Purified GAG fractions were used to quantify mono-sulfated KS (Mono-KS) following established protocols [11, 33, 34]. Frozen tissues (30–50 mg) were homogenized in cold acetone using an Omni Bead Ruptor Bead Mill Homogenizer (OMNI International, Kennesaw, GA) and centrifuged at 12,000 × *g* for 30 min at 4 °C. Defatted pellets were dried, incubated with 0.5 M NaOH for 2 h at 50 °C, neutralized with 1 N HCl, and adjusted to 3 M NaCl. After removing nucleotides by centrifugation, proteins were precipitated by acidification (pH < 1), and GAGs were reprecipitated using ethanol with potassium acetate. Dried pellets were dissolved in 50 mM Tris-HCl (pH 7.0) and analyzed through LC-MS/MS according to the previous protocol [11, 33, 34].

### Assessment of bone and heart pathology

Hearts and knee joints were fixed in 4% PFA. Joints were decalcified in 10% EDTA for 3–4 weeks; hearts were processed directly. Samples were rinsed, dehydrated through a graded ethanol series, and embedded in JB-4 resin (JB-4 Plus Embedding Kit, EMS, Hatfield, PA). Sections (0.5–1.0 µm) were cut on an ultramicrotome, mounted on Superfrost Plus slides, stained with 1% toluidine blue (TB), rinsed, dried, and coverslipped, as described previously [10, 12]. Toluidine blue (TB)–stained knee and heart sections were scored for structural abnormalities using a 0–3 severity scale (0 = normal, 1 = mild, 2 = moderate, 3 = severe) based on the extent of vacuolization and disruption of normal tissue architecture (Supplementary Tables S2–S3). Growth plate scoring assessed architectural disruption, storage vacuoles, and zonal organization; meniscal and ligamentous tissues were evaluated for matrix and cellular changes. Heart sections were examined for vacuolization, cardiomyocyte organization, and matrix expansion. All scoring was performed by a single reviewer in triplicate, blinded to genotype classification. Chondrocyte size was measured in ~100 proliferative‑zone cells per slide.

Sections were imaged on an Olympus BX63 microscope (20×–40×). Images were processed in ImageJ/Fiji (version 1.53; NIH, Bethesda, MD, USA) for qualitative and quantitative evaluation.

### Micro-computed tomography (micro-CT) analysis

Femora were dissected, stored in ethanol, and rehydrated before scanning using the Bruker SkyScan^®^ 1276 system (Bruker, Manning Park, MA) at a voxel size of 8.0 μm, with an X-ray source voltage of 85 kV and current of 47 μA. as described previously [34–36]. Reconstructions (NRecon) and morphometric analyses (CTAn; v1.17.7.2; Bruker) assessed trabecular bone volume (BV/TV, %), trabecular thickness (Tb.Th, mm), trabecular separation (Tb.Sp, mm), cortical thickness (Ct.Th, mm), and bone mineral density (BMD, gHA/cm³). Thresholding was optimized to ensure consistent grayscale calibration. Visualization was performed in CTVol (v2.2.1; Bruker).

### Mechanical testing

Humeri collected at 16 weeks were stored at −80 °C and thawed in PBS before three-point bending. Bones were loaded at 0.1 mm/s with a 10 mm span and 0.1 N preload. Testing was performed on an Instron 5848 MicroTester (Instron, Norwood, MA). Load-displacement curves were recorded to extract mechanical parameters. Biomechanical testing was performed in a limited exploratory cohort (*n* = 3 per genotype) and should be interpreted accordingly.

### Computational analysis of GALNS

Using the AlphaFold 3 online software (https://alphafoldserver.com/), we predicted the possible three-dimensional structures of three mouse GALNS proteins: WT, p.R384C, and p.R384Y variants [37]. We defined Chain A as the amino-acid sequence excluding the 23-amino-acid signal sequence on the N-terminus. One calcium ion was entered as chain B, and then structural models were made as monomers. After obtaining five possible structures for each GALNS, we evaluated the predicted template modeling (pTM) score and the mean predicted aligned error (PAE) from residue 384 to all other residues. We also evaluated the Cα predicted local distance difference test (pLDDT) of residue 384.

### Statistical analysis

Statistical analyses were conducted in GraphPad Prism (version 10.6.1). Normality was assessed using the Shapiro-Wilk test. Parametric data were analyzed using one-way ANOVA with Tukey’s post hoc test, and nonparametric data were analyzed using Kruskal-Wallis with Dunn’s corrections. Significance was set at *p* < 0.05.

Multivariate analyses, including Principal Component Analysis (PCA), were conducted in R (version 4.4.1), GraphPad Prism, and Python (version 3.11) to integrate datasets and identify cross-parameter relationships. PCA was applied as a data integration tool, not to resolve variant-specific phenotypes. PCA was used to reduce dimensionality and assess clustering patterns among genotypes and tissues- i.e., specifically to integrate biochemical and skeletal parameters and to identify independent axes of variation across samples. Before PCA, all quantitative variables were mean-centered and scaled to unit variance. Analyses were conducted using the R package ggplot2. The first two principal components, which together accounted for the majority of the total variance, were visualized in score plots, with 95% confidence ellipses showing group separation. Variable loadings were examined to identify features contributing most strongly to each component.

Force–displacement data were processed in Python to compute stiffness, yield point, and energy absorption.

## Results

### Establishment and genotypic validation

Experimental cohorts were derived from an established GALNS-deficient mouse line carrying the R384C variant, which had been maintained over multiple generations. Initial cohort assignment was based on PCR genotyping. To confirm cohort suitability before downstream analyses, baseline validation was performed by assessing genotype–phenotype concordance using GALNS enzyme activity and toluidine blue–stained histopathology.

**Fig. 1:**
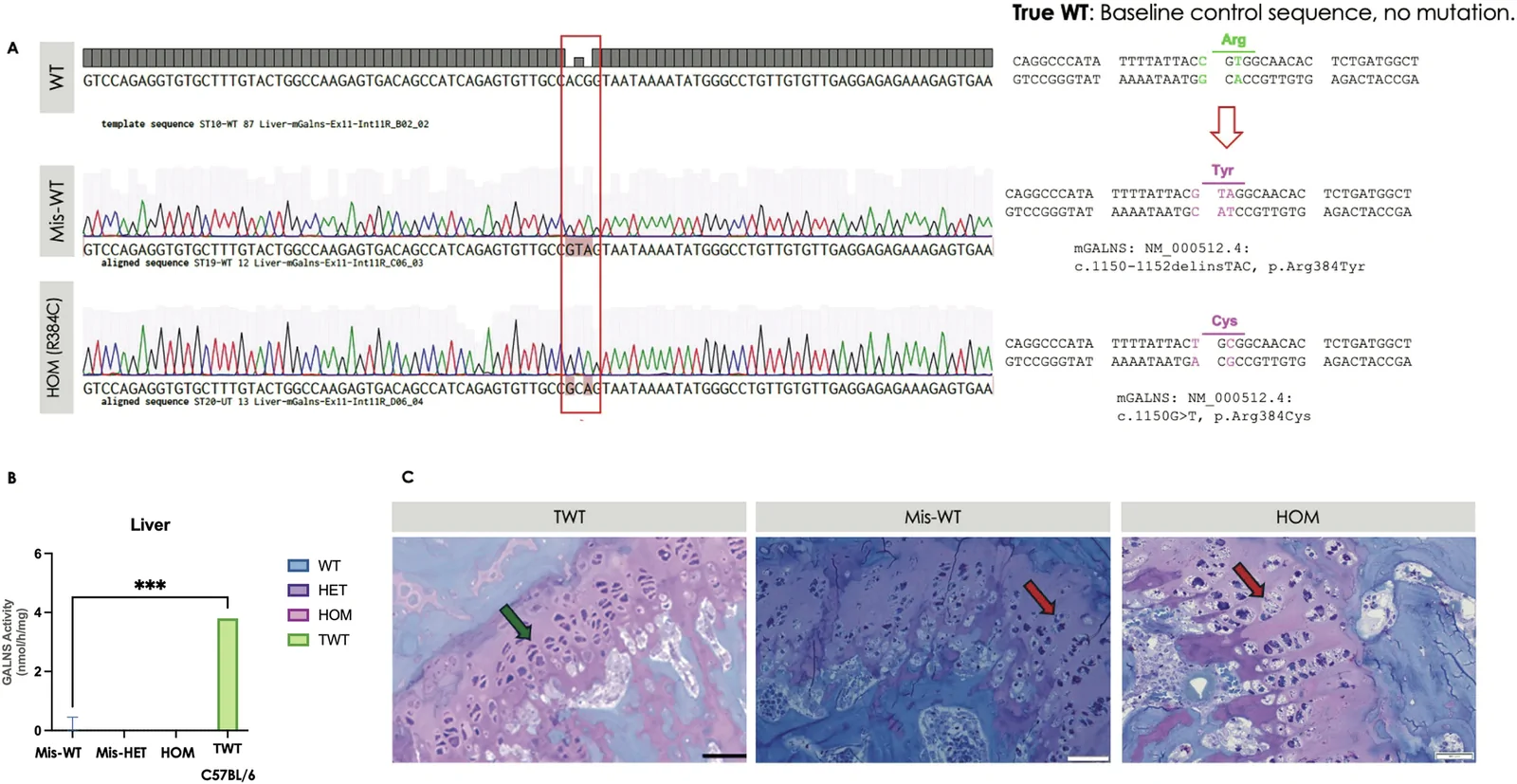
Establishment and baseline validation of experimental cohorts. **A** Molecular confirmation of samples. Sanger chromatograms for WT, R384C, and R384Y alleles. **B** GALNS enzyme activity comparison among WT, R384C, and R384Y samples. **C** Representative histological differences among true WT, R384C, and R384Y mice. Green arrows indicate the absence of vacuolization, while red arrows indicate vacuolization in chondrocytes

A subset of mice classified as WT exhibited markedly reduced GALNS activity, comparable to that of homozygous mutants, and displayed characteristic lysosomal vacuolation and matrix disruption (Fig. 1B, C). Discordant interrogation prompted genotypic reassessment using Sanger sequencing of the *Galns* locus revealing two segregating alleles, R384C and R384Y (Fig. 1A), within the colony lineage, each corresponding to loss-of-function variants and supporting refinement of experimental cohort classification. Both alleles were transmitted through the germline across generations and demonstrated stable segregation following backcrossing (Supplementary Figure S2), reducing concern for persistent founder mosaicism. No new alleles were detected after line establishment (Fig. 1A). Based on these observations, mice were reclassified into two mutant cohorts along with WT controls. The R384Y cohort was retained to facilitate the evaluation of potential variant-specific biological effects. To distinguish the R384C and R384Y variants and to eliminate any additional unintended variants, we devised the scheme depicted in Supplementary Fig. S2. Additional PCR-RFLP assays, WT-sequence/site confirmation, and intronic flanking PCR spanning exon 11 supported integrity of the evaluated local genomic interval surrounding the edited locus (Supplementary Fig. S2).

Heterozygous mice (carriers) were used primarily for breeding and genotyping validation; consistent with carrier status, enzyme activity was intermediate between WT and homozygous mutants (Supplementary Fig. S2). No disease-relevant histopathology in heterozygotes was expected or observed.

### Body-weight trajectories

To evaluate overall physiological impact, body weight was measured weekly from 4 to 16 weeks of age in WT, *Galns*^*R384C*^, and *Galns*^*R384Y*^ mice. Body weight served as a non-invasive longitudinal indicator of growth and systemic health. Mean body-weight trajectories (Supplementary Fig. S3) increased steadily in WT mice, consistent with expected C57BL/6J growth patterns. Both *Galns*^*R384C*^ and *Galns*^*R384Y*^ mice exhibited body weight trajectories comparable to those of age-matched WT controls.

### Biochemical analyses

#### Enzyme assay

**Fig. 2:**
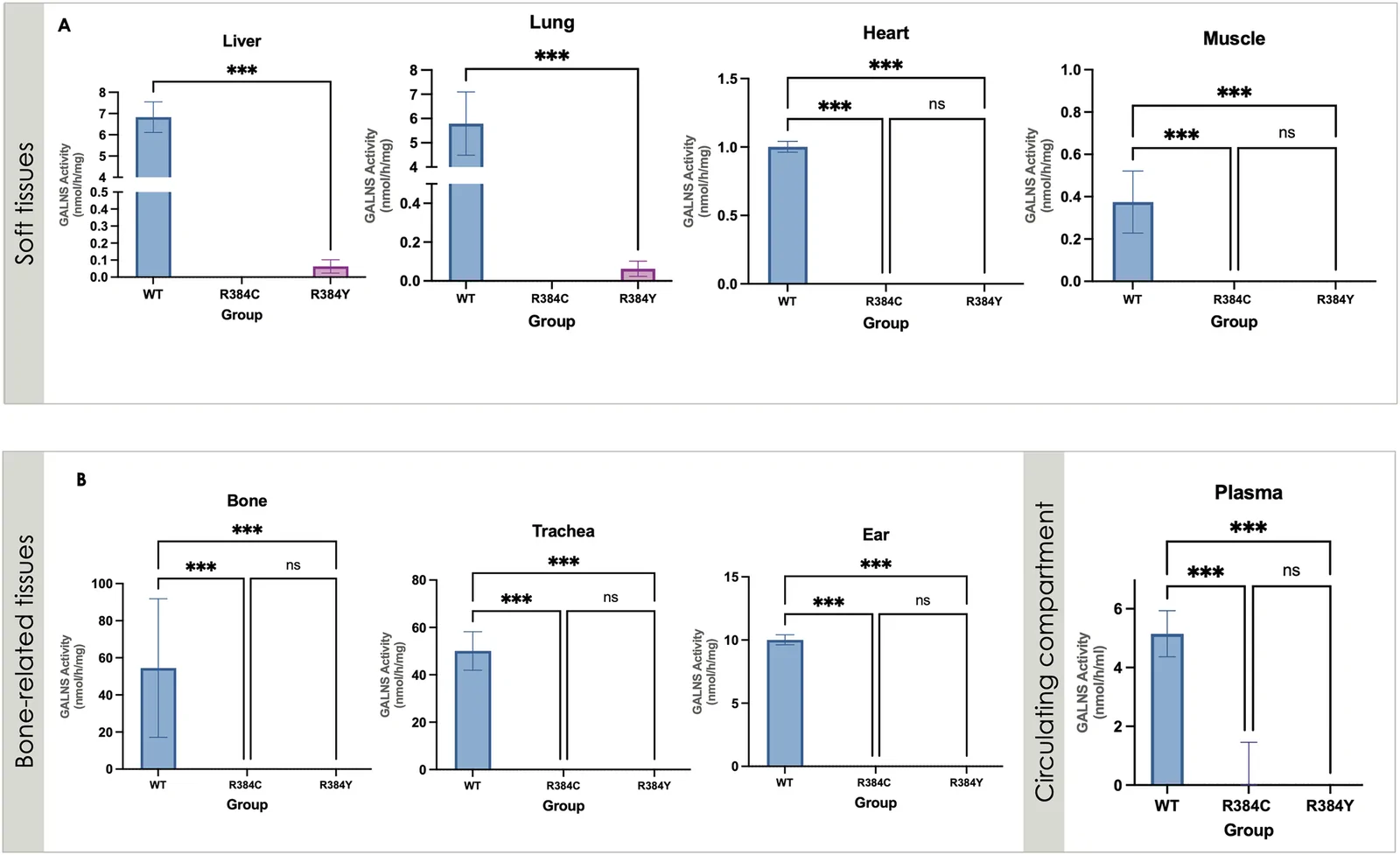
Biochemical characterization of GALNS enzyme. **A** GALNS-specific activity (nmol 4-MU h⁻¹ mg⁻¹) in soft tissues of WT, R384C, and R384Y groups. **B** GALNS-specific activity in bone-related tissues and the circulating compartment (bone, trachea, ear, and plasma), showing residual enzyme activity distribution across structural and systemic sites. Error bars represent mean ± SD (*n* = 4–12 per group); *p < 0.05, **p < 0.01, ***p < 0.001

GALNS enzymatic activity was assessed in plasma and tissue samples (Fig. 2). Both the *Galns*^*R384C*^ and *Galns*^*R384Y*^ mouse models demonstrated no detectable or only minimal GALNS activity in plasma, liver, lung, heart, muscle, bone, trachea, and ear. In WT mice, GALNS activity was observed in liver, lung, heart, muscle, bone, trachea, and ear tissues at levels ranging from 0.37 (muscle) to 54.5 (bone) nmol/h/mg, and in plasma at 5.2 nmol/h/ml on average.

Collectively, these results confirmed a biochemical phenotype consistent with MPS IVA, with both R384C and R384Y variants causing loss-of-function of GALNS, and R384C serving as the primary disease model.

#### Keratan sulfate

**Fig. 3:**
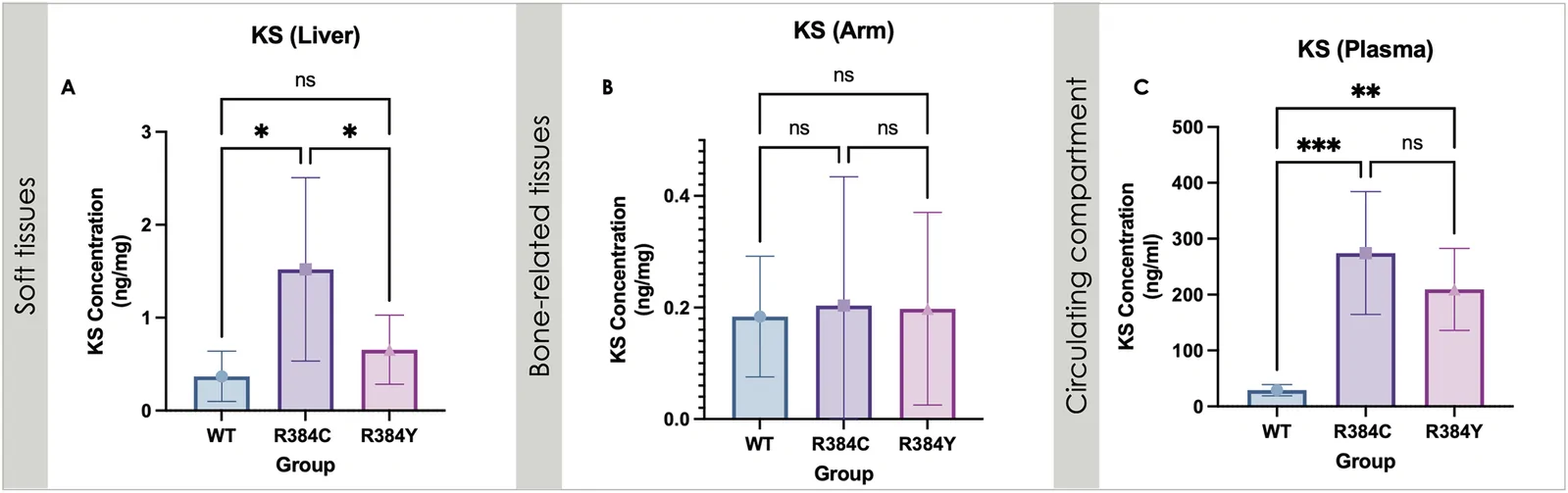
Biochemical characterization of GAG accumulation. Total GAG concentration (μg GAG mg⁻¹ protein) in (**A**) soft tissues, (**B**) bone-related tissues, and (**C**) plasma, demonstrating compartment-specific substrate accumulation. Error bars represent mean ± SD (*n* = 4–12 per group); * *p* < 0.05, ** *p* < 0.01, *** *p* < 0.001

To evaluate the impact of loss-of-function variants on GAG turnover, KS levels were measured by LC–MS/MS in liver, bone (arm), and plasma samples obtained from 16-week-old mice (Fig. 3). In soft tissues, *Galns*^*R384C*^ mice demonstrated significantly elevated KS levels (~4.13-fold compared to wild type, *p* < 0.05), indicative of reduced substrate turnover, while *Galns*^*R384Y*^ mice exhibited a modest, non-significant increase. In bone-related tissues, KS accumulation was minimal in both mutant groups, with *Galns*^*R384Y*^ mice showing a 1.78-fold increase relative to WT (not statistically significant), a pattern similarly noted in *Galns*^*R384C*^ mice. Conversely, plasma KS levels were increased, with *Galns*^*R384C*^ displaying around a 9.41-fold rise over WT (*p* < 0.01), and *Galns*^*R384Y*^ mice exhibiting a 7.18-fold increase (*p* < 0.0001), indicative of systemic overflow of accumulated substrates.

### Histopathology

**Fig. 4:**
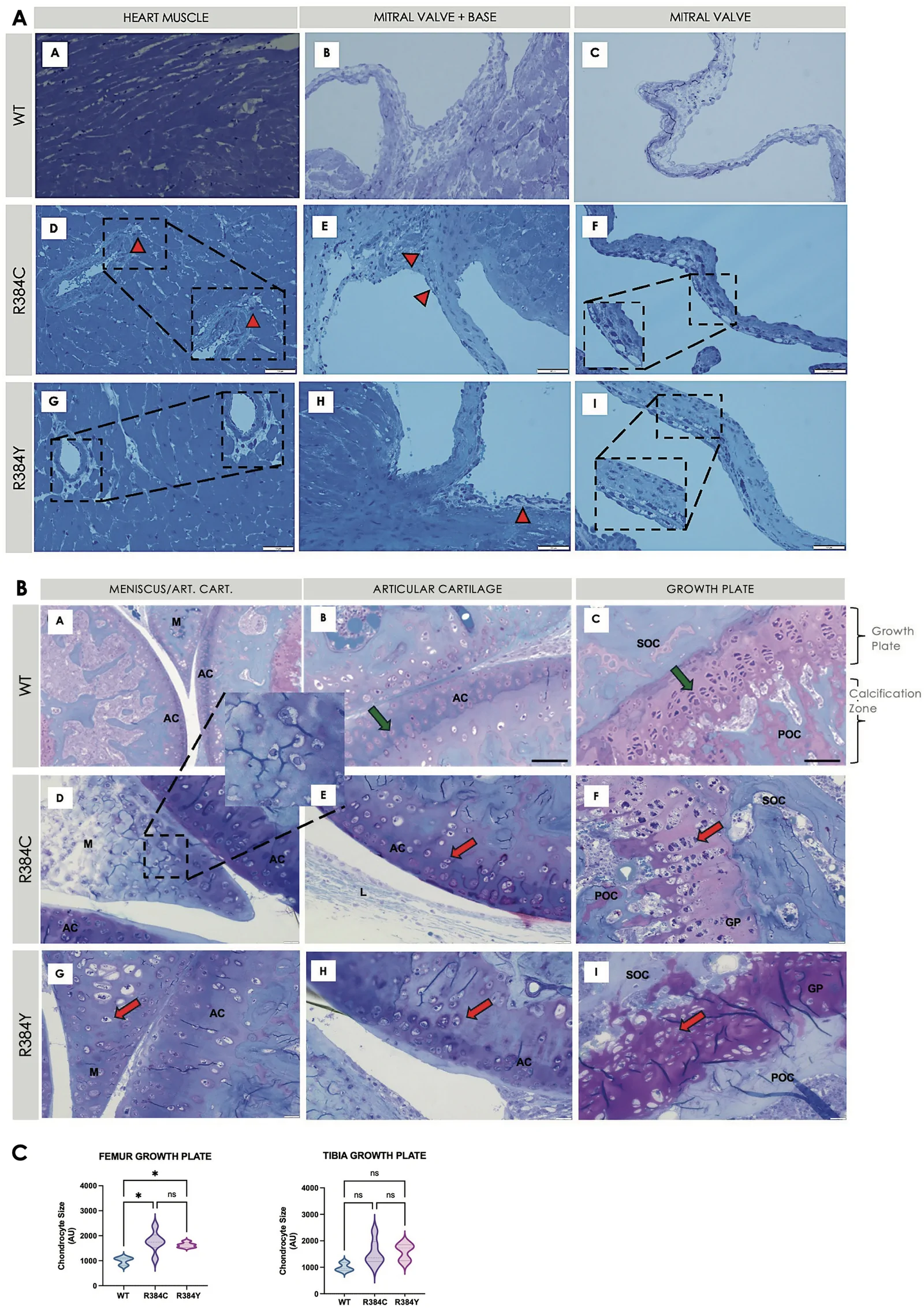
Representative Skeletal and Cardiac Pathology with Chondrocyte Morphometry. **A** Histological evaluation of coronal heart sections stained with toluidine blue from WT (**A**–**C**), R384C (**D**–**F**), and R384Y (**H**–**I**) mice (16-week-old). Representative images of the heart muscle, mitral valve, and the base of the mitral valve are shown. Arrows and inset highlights show vacuolization (deposition of KS/C6S) in cells. **B** Histological evaluation of sagittal knee joint sections stained with toluidine blue from WT (**A**–**C**), R384C (**D**–**F**), and R384Y (**H**–**I**) mice (16 weeks old). Representative images of the meniscus, articular cartilage, and growth plate are shown, with the primary ossification center (POC) and secondary ossification center (SOC) indicated to orient the anatomical context of the growth plate. Scale bars: 100 mm. **C** Chondrocyte cell size within the proliferative zone of the epiphyseal growth plate. Violin plots show chondrocyte size (arbitrary units per 100 cells) quantified from toluidine blue–stained JB-4 growth plate sections

Histological analyses were performed to assess tissue-level pathology resulting from loss-of-function variants, focusing on the knee joint and heart tissues commonly affected in MPS IVA (Fig. 4). The knee joint was examined to evaluate growth-plate vacuolization, organization, and chondrocyte morphology, while the heart was analyzed for valvular and myocardial vacuolization.

In the heart, WT mice showed that mitral valves were thin and cardiac tissues lacked cytoplasmic vacuolization, indicating normal cardiac architecture. In contrast, both *Galns*^*R384C*^ and *Galns*^*R384Y*^ mice exhibited marked valvular thickening and prominent cytoplasmic vacuoles consistent with lysosomal storage in the mitral valve, myocardium, and pericardium (Fig. 4A, Supplementary Fig. S4). Semiquantitative scoring confirmed a significant increase in vacuolization, compared with WT mice (*p* < 0.001), with no significant difference between R384 C and R384Y groups (Supplementary Table S2).

Knee joint analysis revealed distinct genotype-dependent abnormalities. WT mice exhibited an organized columnar structure of chondrocytes, devoid of vacuolization, with preserved zonal architecture and consistent toluidine blue metachromasia. Conversely, both *Galns*^*R384C*^ and *Galns*^*R384Y*^ murine models demonstrated disrupted columnar organization, extensive chondrocyte vacuolization, and irregular loss of metachromasia, suggestive of excessive glycosaminoglycan accumulation and compromised matrix turnover (Fig. 4B). Semiquantitative scoring indicated a significant increase in vacuolization and growth plate disorganization in both mutant groups compared to WT mice (*p* < 0.01; Supplementary Table S3). Vacuolization was corroborated in chondrocytes within the meniscus and ligaments of mutant mice (Fig. 4B).

Quantitative analysis indicated that there is a significant expansion of proliferative zone chondrocytes in both *Galns*^*R384C*^ and *Galns*^*R384Y*^ mice relative to WT controls (*p* < 0.05). No significant difference was observed between the mutant groups (Fig. 4C). Collectively, these findings demonstrate marked skeletal and cardiac pathology in both models, with *Galns*^*R384Y*^ mice displaying a disease phenotype comparable to that of *Galns*^*R384C*^ mice.

### Skeletal imaging

Micro-CT was employed to evaluate the skeletal microarchitecture in the femur, a reproducible and biomechanically significant long bone, to ascertain whether biochemical and histopathological abnormalities corresponded to structural alterations and whether *Galns*^*R384Y*^ mice grouped with *Galns*^*R384C*^ mice rather than with WT mice.

**Fig. 5:**
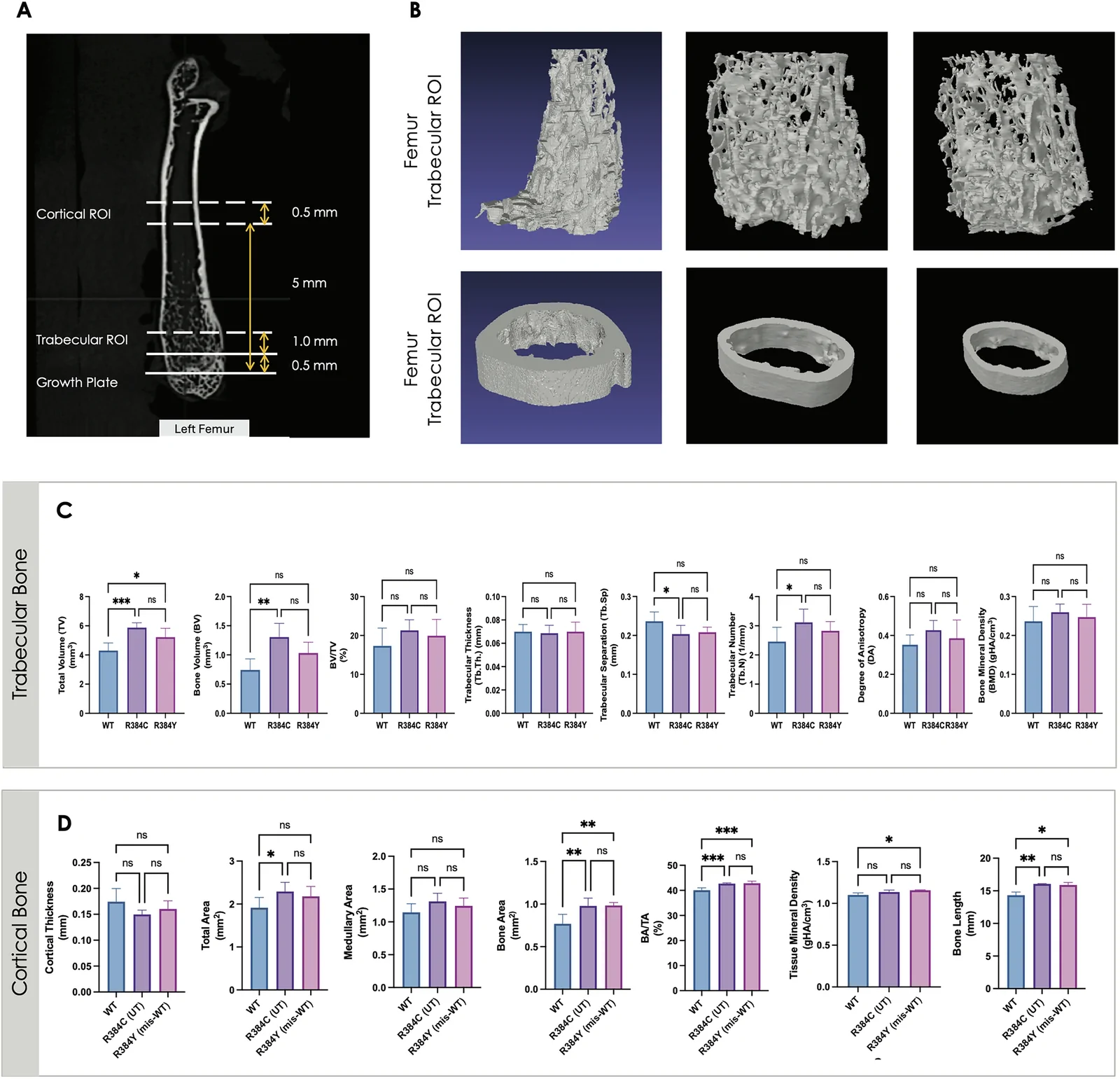
Micro-CT analysis of femoral bones obtained from 16-week-old mice. **A** A representative image of the femur illustrating the regions of interest (ROIs) for the analysis of trabecular and cortical bone. **B** Representative cross-sectional images from WT mice (*n* = 5), R384C mice (*n* = 7), and R384Y mice (*n* = 5). Quantitative analysis derived from the micro-CT scans of the (**C**) femur trabecular and (**D**) femur cortical bone. The graphs depict the mean ± standard deviation (SD): one-way ANOVA with Tukey’s multiple comparison test; *p < 0.05, ** *p* < 0.01, *** *p* < 0.001, and **** *p* < 0.0001. “ns” indicates not statistically significant

Trabecular analysis revealed genotype-dependent alterations in metaphyseal architecture (Fig. 5). Total trabecular volume was significantly increased in both *Galns*^*R384C*^ (*p* < 0.001) and *Galns*^*R384Y*^ mice (*p* < 0.05) relative to WT. *Galns*^*R384C*^ mice exhibited additional increases in trabecular bone volume and trabecular number, along with reduced trabecular separation, indicating denser but abnormal trabeculation. In contrast, *Galns*^*R384Y*^ mice showed metaphyseal expansion without significant changes in trabecular organization.

Cortical assessment at the mid-diaphysis demonstrated significant increases in bone area and bone area fraction in both mutant groups compared with WT (*p* < 0.001), with BA/TA increased by ~27–28% (Fig. 5). Tissue mineral density trended higher in *Galns*^*R384Y*^ mice but did not differ significantly in *Galns*^*R384C*^ mice. Femoral length was increased in both mutant groups despite no difference in body weight at 16 weeks.

Overall, *Galns*^*R384Y*^ mice phenocopied *Galns*^*R384C*^ mice across cortical measures, indicating that both missense variants are sufficient to disrupt cortical bone modeling and drive skeletal changes characteristic of untreated MPS IVA. Biochemical substrate (KS) accumulation in bone and skeletal microarchitecture did not show linear correspondence, consistent with prior murine MPS IVA studies.

### Biomechanical testing

Three-point bending tests were conducted on the humerus to assess whether microarchitectural changes detected by micro-CT translated into variations in mechanical performance. Force–displacement curves were generated for WT, *Galns*^*R384C*^, and *Galns*^*R384Y*^ mice, and parameters such as ultimate force, stiffness, work to fracture, and bending rigidity (EI) were calculated to incorporate both material and geometric contributions.

**Fig. 6:**
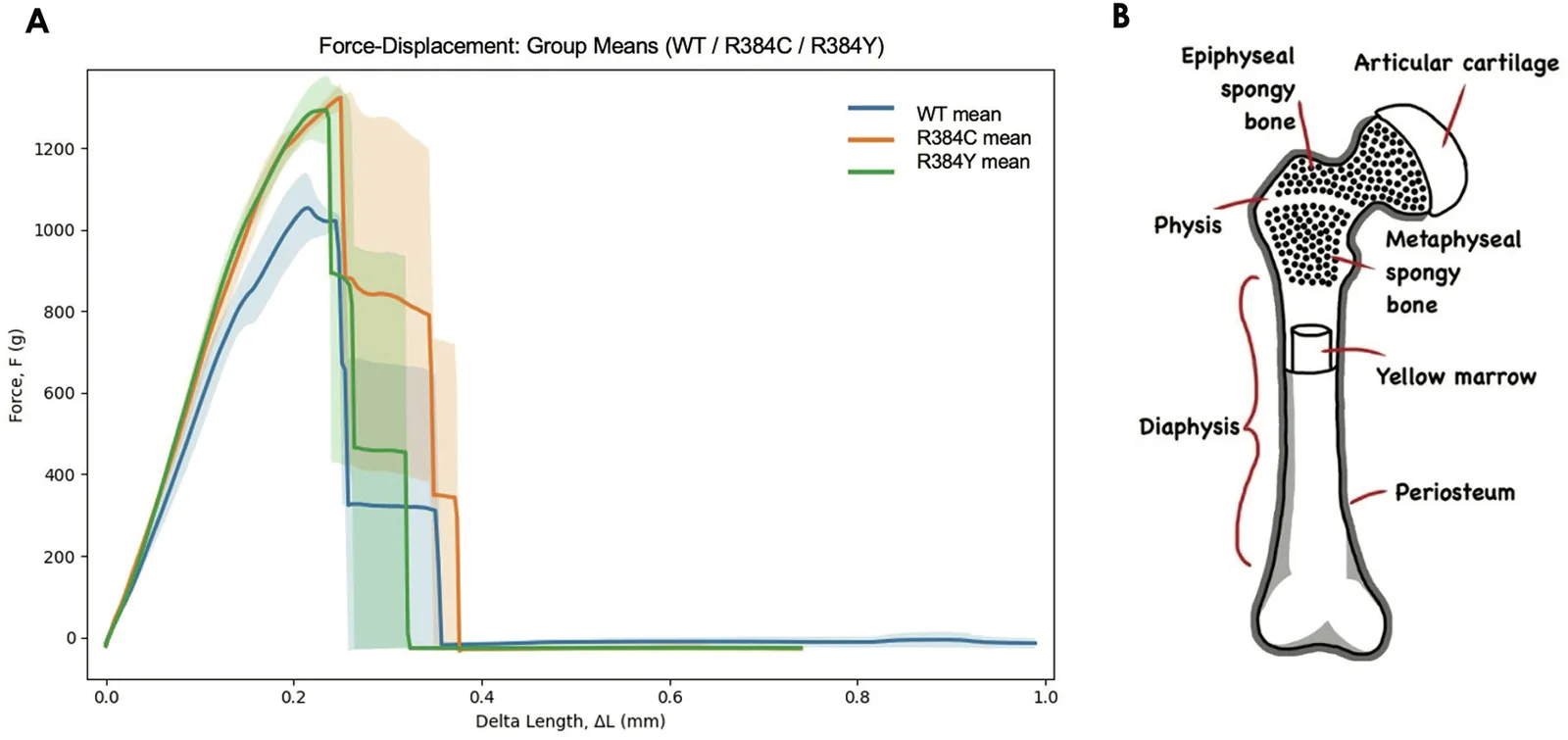
Mechanical properties of humeri shafts assessed by three-point bending tests. **A** Mean force–displacement (F–D) curves for WT (blue), R384C (orange), and R384Y (green) groups demonstrate comparable overall mechanical response patterns across genotypes. **B** Schematic of long bones showing the metaphyseal and epiphyseal regions mainly affected by disrupted chondrocyte organization

Across all parameters, no statistically significant differences were observed among genotypes (Supplementary Fig. S5). Mean force–displacement curves showed comparable elastic and post-yield behavior, with overlapping ultimate force, stiffness, and energy absorption values. Minor visual differences in force–displacement curves were inconsistent across specimens and were not quantified (Fig. 6). Biomechanical testing was included to evaluate whether early microarchitectural changes translated into gross mechanical deficits at this age; the absence of differences supports the compartment-specific nature of early pathology.

### Multivariate analyses

PCA was performed to integrate biochemical measures (GALNS activity and GAG levels) with bone morphometric parameters, including Ct.Th, Tb.Th, BMD, and BA/TA, and to examine multivariate relationships among genotypes (see Supplementary Fig. S6). The first principal component (PC1) explained 46.9% of the total variance and captured an inverse relationship between GALNS activity (negative loading) and GAG accumulation (positive loading), reflecting the primary enzyme–substrate axis. The second principal component (PC2) accounted for 24.3% of the variance and was driven by skeletal structural parameters, including Ct.Th, Tb.Th, and BMD.

PCA was used as an integrative, hypothesis-generating approach to assess the convergence of biochemical and skeletal phenotypes across genotypes rather than to delineate variant-specific subgroups. Clear separation between WT and mutant groups was observed along PC1, driven by differences in GALNS activity and substrate accumulation, whereas *Galns*^*R384C*^ and *Galns*^*R384Y*^ mice exhibited substantial overlap. This pattern indicates phenotypic convergence across distinct missense variants at the multivariate level, supporting their classification as comparably severe disease phenotypes. Along with PC2, both mutant groups were displaced relative to WT in a manner consistent with altered bone microarchitecture, with *Galns*^*R384Y*^ clustering closely with *Galns*^*R384C*^. Multivariate group separation was statistically supported by PERMANOVA, confirming significant differences between WT and both mutant groups, while R384C and R384Y did not differ significantly from each other (Supplementary Table S4 and Supplementary Fig. S6). Together, these analyses indicate that the two mutant genotypes occupy overlapping multivariate spaces under the conditions examined, with differences insufficient to support their discrimination as distinct phenotypic subgroups.

### Computational analysis of murine GALNS (simulated structural modeling)

A graphical summary and condensed tabulation of these prediction metrics are shown in Supplementary Fig. S7, while complete model-specific values are provided in Supplementary Table S5. The pTM scores for all models were 0.96 or 0.97, suggesting that the accuracy of the predictions’ global structure is very high. Local confidence at residue 384 (mean Cα pLDDT) was reduced in both variants, with a more pronounced decrease in R384Y (97.5 in R384C and 92.5 in R384Y; from 98.8 in WT). PAE analyses showed increased positional uncertainty from residue 384 to the rest of the residues (from 1.80 Å in the WT to 2.47 Å in R384C and 5.03 Å in R384Y).

These results indicate that the local uncertainty around residue 384 is greater in these variant proteins than in the WT, especially in the R384Y variant [37], implying that the local structure around residue 384 may be less rigid in these two variants.

## Discussion

MPS IVA is a genetically heterogeneous lysosomal storage disorder in which disease severity is most often driven by missense mutations in the *GALNS* gene rather than truncating variants [16, 17]. Among these, p.R386C (p.R384C in mice) is one of the most frequently reported pathogenic alleles in patient cohorts and is commonly associated with severe disease [17, 22, 25]. The high prevalence of compound heterozygosity complicates genotype–phenotype inference, underscoring the need for controlled experimental systems carrying clinically relevant missense variants [16, 38–40].

To address this gap, we generated a knock-in mouse model harboring the patient-relevant R384C missense substitution. During CRISPR/Cas9-mediated genome editing, an unintended second variant, R384Y, emerged and was stably propagated following segregation by backcrossing. This allele was retained to examine whether distinct missense substitutions at the same residue yield divergent or convergent phenotypic outcomes within a uniform genetic background.

Across biochemical, histological, skeletal, and multivariate analyses, both *Galns*^*R384C*^ and *Galns*^*R384Y*^ mice exhibited a severe MPS IVA phenotype consistent with previously established murine models [12–15, 21, 33, 34, 41]. GALNS activity was near-undetectable in both mutant cohorts, accompanied by elevated KS levels in plasma and soft tissues and marked lysosomal vacuolization in cartilage and cardiac tissues. Enzyme activity in WT mice followed expected tissue-specific patterns, whereas both mutant cohorts showed a profound loss of activity across the tissues examined [13, 14, 33]. Bone KS levels remained low, diverging from the inverse activity–substrate relationship observed in soft tissues and plasma, consistent with previous rodent studies and reflecting species-specific differences in cartilage composition relative to humans, in whom bone and cartilage represent major KS-rich compartments [18, 42, 43].

Histopathological evaluation demonstrated extensive chondrocyte vacuolization and disrupted columnar organization of the growth plate in both mutant groups, accompanied by prominent cardiac valve pathology. Micro-CT analyses further revealed comparable alterations in trabecular and cortical bone architecture, indicating that both variants were sufficient to disrupt normal endochondral bone modeling. Multivariate integration of biochemical and skeletal datasets demonstrated clear separation between WT and mutant mice and substantial overlap between R384C and R384Y, supporting phenotypic convergence rather than variant-specific divergence. These results were also consistent with the previously established MPS IVA mouse models [12–15, 21, 33, 34, 41]. We clarified that biochemical substrate burden and skeletal architecture do not necessarily correlate linearly.

Despite marked biochemical and microarchitectural abnormalities, biomechanical testing did not reveal significant differences in whole-bone mechanical performance at the age examined. This dissociation suggests that early skeletal pathology in this model primarily affects growth plate and trabecular compartments before manifesting as measurable deficits in diaphyseal bone strength, consistent with compartment- and stage-dependent progression in MPS IVA [38].

Structural considerations provide a plausible mechanistic basis for the observed phenotypic convergence. Arg384 of mouse N-acetylgalactosamine-6-sulfate sulfatase resides at the rim of the GALNS active-site cavity, based on the computationally predicted model on the Protein Data Bank (https://www.rcsb.org/structure/AF_AFQ571E4F1), and contributes to local electrostatic stability. Substitution with either cysteine or tyrosine may disrupt local electrostatic interactions and may compromise protein stability rather than directly impair catalytic residues. These interpretations are based on existing structural data and should be regarded as hypothesis-generating, as direct assessment of protein folding, trafficking, or degradation was not performed.

However, exploratory computational analyses using AlphaFold suggest that both R384C and R384Y variants largely preserve global protein folding, as reflected in pTM scores relative to WT. On the other hand, variants at residue 384 appeared more responsive to changes in predicted local confidence and inter-residue positional uncertainty [as described in the results]. These observations suggest that although the overall fold is maintained, perturbations of long-range structural coordination, potentially influencing domain interactions or active-site geometry, could have a greater effect - i.e., local structure around residue 384 might be less rigid in these two variants. Importantly, these computational predictions do not directly demonstrate altered protein folding and should be interpreted as hypothesis-generating, highlighting regions that warrant experimental investigation.

The ability of both R384C and R384Y substitutions to recapitulate a shared disease phenotype supports a threshold-based model in which disease manifestation is governed primarily by the degree of GALNS impairment rather than the precise amino-acid substitution. Once residual activity falls below a critical functional threshold, distinct missense variants may converge on a common pathological spectrum, thereby providing a framework for contextualizing genotype–phenotype variability in patients.

The presence of multiple mutant alleles within the engineered lineage is consistent with known outcomes of CRISPR/Cas9 genome editing [28, 44]. In this case, stable propagation of both variants enabled additional comparative analyses within a controlled genetic background. Stable propagation of both variants enabled direct evaluation of phenotypic convergence within a controlled genetic background, eliminating confounding effects inherent to human heterogeneity. These findings provide a rationale for future intervention studies to evaluate whether increasing residual GALNS activity above a functional threshold modifies pathology; however, therapeutic responsiveness cannot be inferred from the current characterization study.

Several limitations should be noted. First, analyses were limited to male mice with modest cohort sizes. Assessment of sex-specific effects was beyond the scope of the present study and warrants future investigation. Second, the emergence of a secondary variant after genome-editing highlights the importance of rigorous allele-level validation in CRISPR-generated models. Third, although complementary molecular approaches, including PCR-RFLP genotyping, WT-sequence/site confirmation, intronic amplification spanning exon 11, and Sanger sequencing across *Galns* exons, supported the refinement of genotype classification and the evaluation of the local edited interval following the emergence of an additional allele, long-read sequencing or broader structural genomic analyses were not performed. Therefore, rare complex on-target rearrangements outside the amplified regions cannot be completely excluded. Future studies incorporating broader genomic characterization and additional functional analyses may further strengthen understanding of allele-specific effects and model stability.

Fourth, while computational and molecular findings supported loss-of-function effects, direct experimental assessment of protein folding, trafficking, or degradation was not undertaken. Future studies may further clarify allele-specific mechanisms underlying enzyme dysfunction. Fifth, functional outcomes such as motor performance were not assessed; however, considering our previous models, all MPS IVA mice reflect normal neurological development [2, 3]. Finally, the computational analysis performed by AlphaFold 3 has limitations. All predictions were run with templates enabled, which makes the models somewhat biased toward preserving the known GALNS fold in humans. This makes the models useful for comparing local repacking; however, it does not mean that large fold changes cannot occur. In the case of the p.R384C variant, the risk of incorrect disulfide bonds should not be overlooked [45].

In summary, this work provides the first comprehensive in vivo characterization of a patient-relevant *GALNS* missense mutation in a mouse model and establishes a genotype-to-phenotype framework for MPS IVA. The phenotypic convergence observed between R384C and R384Y mice indicates that severe reduction of GALNS activity, irrespective of the specific missense substitution at this residue, is sufficient to drive a shared pathological spectrum. Extrapolation to therapeutic responsiveness will require direct intervention studies and should be interpreted cautiously.

## Supplementary information

Supplementary
