# Supplementary material for: Comprehensive characterization and translational implications of the GalnsR384C mouse model of Mucopolysaccharidosis IVA
Source: J Hum Genet. 2026 Aug 13;71(11):719–29. doi: 10.1038/s10038-026-01501-x (PMC13695577; doi:10.1038/s10038-026-01501-x)
Supplement: Supplementary file 1 [file 10038_2026_1501_MOESM1_ESM.docx]

**Supplementary Information**

**Comprehensive Characterization and Translational Implications of the *Galns^R384C^* Mouse Model of Mucopolysaccharidosis IVA**

Dione A. Holder^1,2^, Betul Celik^2^, Sampurna Saikia^1,2^, Shaukat Khan^2^, Yasuhiko Ago^2^, Amali Karunathilaka^1,2^, Shunji Tomatsu^1,2^

**Author Affiliation**:

^1^ Department of Biological Sciences, University of Delaware, Newark, DE 19716, USA

^2^ Department of Biomedical Research, Nemours Children’s Health, Wilmington, DE 19803, USA

***Address for correspondence**:

Shunji Tomatsu, MD, Ph.D.

DuPont Experimental Station, Bldg. E400 #5205, 200 Powder Mill Rd. Wilmington, DE 19803, Office Tel: 302-298-7336, stomatsu@nemours.org.

**Supplementary Figure S1**. (A) Schematic representation of the mouse Galns model used in this study. The targeted Exon 11 is highlighted, indicating the location of the c.1152C>T (p.Arg384Cys) substitution introduced by CRISPR/Cas9-mediated genome editing. Exonic boundaries, coding regions, and the precise nucleotide change are shown to illustrate the genomic context of the engineered allele. (B) Experimental Design: Timeline and workflow illustrating group allocation, longitudinal body-weight monitoring, and endpoint assays. Molecular (PCR/Sanger), biochemical (enzyme, GAG, protein), histological (cartilage, heart), micro-CT, and biomechanical analyses were performed sequentially.

**Supplementary Figure S2.** **Extended establishment and baseline validation of experimental cohorts - resolution of misclassification.** (A) Representative PCR genotyping gel showing expected WT, HET, and HOM patterns. (B) Complementary WT-sequence/site assay used to assess retention or loss of the WT allele pattern. Also represented, PCR amplification using intronic flanking primers (Intron10F/Intron11R) spanning exon 11, supporting integrity of the local genomic interval surrounding the edited site. (C) Schematic summarizing how the mosaic founder caused the false WT call. (D) Observed progeny were 100% HET (n = 14), consistent with expectation. (E) Representative PCR genotyping gel showing expected HET patterns as a result of backcross. (F) HET mice showed enzyme activity between WT and HOM, confirming functional heterozygosity.

**Supplementary Figure S3**. **Weekly body-weight trajectories of *Galns^R384C^* mice.** Mean ± SEM body weights plotted from 4 to 16 weeks of age for WT (blue), R384C (red), and R384Y (green).

**Supplementary Figure S4**. **Extended histological characterization.** Toluidine blue–stained heart sections demonstrating vacuolization within the pericardial membrane and surrounding adipose tissue. Arrows and box highlight represent vacuolization. Scale: 100 mm.

**Supplementary Figure S5**. **Mechanical properties of humeri shafts assessed by three-point bending.** (A) Ultimate force (maximum load at failure), (B) Stiffness (slope of the elastic region of the F–D curve), and (C) Work-to-Fracture (area under the F–D curve) were quantified for each group. (D) EI. No statistically significant differences were detected across genotypes for any parameter, indicating that neither R384C nor R384Y humeri exhibit gross reductions in cortical mechanical performance despite underlying biochemical and histological alterations. Data are presented as mean ± SD (n = 3 per group); ns = not significant.

**Supplementary Figure S6**. **PCA of biochemical and micro-CT parameters and corresponding variable loadings**. (A) Principal component analysis (PCA) of micro-CT parameters (BA/TA, Tb.Th, Ct.Th, and BMD) shows separation among the WT, R384C, and R384Y groups. (B) The corresponding loadings plot shows that BA/TA, BMD, and Tb.Th contribute most strongly to PC1. (C) The biplot shows variable loadings (displayed as arrows), indicating the strength and direction of contributions from enzyme activity, GAG measures, and bone microarchitectural parameters to the PCA components.

**Supplementary Figure S7**. **Structural prediction metrics for WT R384C and R384Y GALNS**. (A) Bar graphs showing mean pTM, pLDDT, and PAE values. (B) Summary Table containing key metrics for each model, mean ± SD.

**Supplementary Table S1.** Primer sequences, PCR conditions, and TauI restriction digest patterns used to distinguish wildtype, heterozygous, and homozygous genotypes.

| Genotyping | Primer sequences  (forward and reverse) | PCR Protocol | Product size |
| --- | --- | --- | --- |
| Allelic discrimination PCR | **Forward primer (R384CF):**  5’-ATTTTCCTGAGCCACACACC-3’  **Reverse primer (R384CR):**  5’-CAACAAAGGGCAAGATCCAT-3’ | **PCR Program:**  • **95 °C for 3 min**  **35 cycles:**  • 95 °C for 45 s  • 55 °C for 1 min  • 72 °C for 2 min  **Final extension:** 72 °C for 5 min  **Hold:** 4 °C | **PCR amplicon:** 439 bp |
| Restriction digestion | **Restriction enzyme:** Tau I  **Recognition site:** 5′-GCSG^C-3′  (S = G or C) | **Digestion conditions:**  • Combine PCR product with:  - 2.0 µL 10× buffer B  - 1.0 µL TauI enzyme  - Nuclease-free water to 7.0 µL total  • **Incubate at 55 °C for 1 hour** | **WT:** 335 + 104 bp **HET:** 335 + 202 + 133 + 104 bp **HOMO:** 202 + 133 + 104 bp |
| Restriction digestion | **Restriction enzyme:** Btg I  **Recognition site:** 5′-GCSG^C-3′  (S = G or C) | **Digestion conditions:**  • Combine PCR product with:  - 2.0 µL 10× buffer B  - 0.5 µL BtgI enzyme  - Nuclease-free water to 7.5 µL total  • **Incubate at 37 °C for 1 hour** | **WT:** 242 + 197 bp **HET:** 439 + 242 + 197 bp **HOMO:** 439 bp |

**Supplementary Table S2.** Histological scoring of vacuolization in heart tissues by anatomical region.

| Anatomical Site | WT | R384C | R384Y |
| --- | --- | --- | --- |
| Base | 0 | 3.0 ± 0.0 | 3.0 ± 0.0 |
| Valve | 0 | 3.0 ± 0.0 | 3.0 ± 0.0 |
| Pericardiac membrane | 0 | 3.0 ± 0.0 | 3.0 ± 0.0 |
| Muscle | 0 | 3.0 ± 0.0 | 3.0 ± 0.0 |

**Supplementary Table S3.** Histological scoring of knee joint cartilage and growth plate regions.

| Anatomical Site | Structure | Finding | WT | R384C | R384Y |
| --- | --- | --- | --- | --- | --- |
| Tibia | Growth plate | Vacuolization | 0.0 ± 0.0 | 3.0 ± 0.0 | 3.0 ± 0.0 |
|  |  | Column structure | 0.0 ± 0.0 | 3.0 ± 0.0 | 3.0 ± 0.0 |
|  | Articular cartilage | Vacuolization | 0.0 ± 0.0 | 3.0 ± 0.0 | 3.0 ± 0.0 |
|  |  | Column structure | 0.0 ± 0.0 | 3.0 ± 0.0 | 3.0 ± 0.0 |
| Femur | Articular cartilage | Vacuolization | 0.0 ± 0.0 | 3.0 ± 0.0 | 3.0 ± 0.0 |
|  |  | Column structure | 0.0 ± 0.0 | 3.0 ± 0.0 | 3.0 ± 0.0 |
|  | Growth plate | Vacuolization | 0.0 ± 0.0 | 3.0 ± 0.0 | 3.0 ± 0.0 |
|  |  | Column structure | 0.0 ± 0.0 | 3.0 ± 0.0 | 3.0 ± 0.0 |
| Ligament | - | Vacuolization | 0.0 ± 0.0 | 3.0 ± 0.0 | 3.0 ± 0.0 |
| Meniscus | - | Vacuolization | 0.0 ± 0.0 | 3.0 ± 0.0 | 3.0 ± 0.0 |

**Supplementary Table S4**. PERMANOVA results comparing multivariate group differences based on biochemical and structural parameters (999 permutations, Euclidean distance).

| Comparison | pseudo-F | p-value | Significance |
| --- | --- | --- | --- |
| WT vs R384C | 10.526 | 0.004 | ** |
| WT vs R384Y | 7.377 | 0.006 | ** |
| R384C vs R384Y | 1.6535 | 0.152 | ns |

**Supplementary Table S5**. **AlphaFold structural confidence metrics for wild-type and GALNS variants (R384C and R384Y)**. AlphaFold-derived metrics indicate preserved global folding and localized uncertainty.

|  | pTM  (Global Fold) | | | | Residue 384 Cα pLDDT  (Local Prediction Confidence) | | | Mean PAE from residue 384 (Å)  (Relative Positional Uncertainty) | | |
| --- | --- | --- | --- | --- | --- | --- | --- | --- | --- | --- |
|  | **WT** | **R384C** | **R384Y** | **WT** | | **R384C** | **R384Y** | **WT** | **R384C** | **R384Y** |
| Model 0 | 0.97 | 0.97 | 0.96 | 98.82 | | 97.66 | 92.32 | 1.802 | 2.412 | 5.019 |
| Model 1 | 0.97 | 0.97 | 0.96 | 98.82 | | 97.59 | 91.9 | 1.797 | 2.425 | 5.26 |
| Model 2 | 0.97 | 0.97 | 0.96 | 98.82 | | 97.45 | 92.6 | 1.79 | 2.494 | 4.803 |
| Model 3 | 0.97 | 0.96 | 0.96 | 98.82 | | 97.45 | 92.74 | 1.823 | 2.479 | 5.051 |
| Model 4 | 0.97 | 0.96 | 0.96 | 98.82 | | 97.26 | 92.74 | 1.815 | 2.529 | 4.993 |
| Average  (5 models) | 0.97 | 0.966 | 0.96 | 98.82 | | 97.482 | 92.46 | 1.8054 | 2.4678 | 5.0252 |

**Abbreviations:** WT, wild-type; pTM, predicted template modeling score; pLDDT, predicted local distance difference test; PAE, predicted aligned error.

pLDDT values at residue 384 reflect local structural confidence. Mean PAE values represent positional uncertainty of residue 384 within the entire structure. Higher values indicate greater uncertainty.
